# Cognitive Impairment in Liver Transplant Recipients With a History of Cirrhosis: A Systematic Review

**DOI:** 10.1097/TXD.0000000000001479

**Published:** 2023-04-20

**Authors:** Osama M. Siddiqui, Archit B. Baskaran, Katherine A. Lin, Naela Najam, Tahir Shah, Molly L. Beestrum, Avesh Thuluvath, Borna Bonakdarpour, Minjee Kim, Zachary Dietch, Michael Wolf, Daniela P. Ladner

**Affiliations:** 1 Northwestern University Transplant Outcomes Research Collaborative (NUTORC), Comprehensive Transplant Center (CTC), Feinberg School of Medicine, Northwestern University, Chicago, IL.; 2 Department of Neurology, University of Chicago Medical Center, University of Chicago, Chicago, IL.; 3 Department of Neuroscience, University of Cincinnati, Cincinnati, OH.; 4 Galter Health Sciences Library, Feinberg School of Medicine, Northwestern University, Chicago, IL.; 5 Division of Gastroenterology and Hepatology, Department of Medicine, Northwestern Medicine, Chicago, IL.; 6 Division of Behavioral Neurology, Department of Neurology, Northwestern University Feinberg School of Medicine, Chicago, IL.; 7 Division of Neurocritical Care, Department of Neurology, Northwestern University Feinberg School of Medicine, Chicago, IL.; 8 Division of Transplant, Department of Surgery, Northwestern Medicine, Chicago, IL.; 9 Division of General Internal Medicine & Geriatrics, Northwestern University Feinberg School of Medicine, Chicago, IL.

## Abstract

Cognitive impairment is common among patients with cirrhosis and may persist post-transplantation. This systematic review seeks to (1) describe the prevalence of cognitive impairment in liver transplant (LT) recipients with a history of cirrhosis, (2) describe risk factors for this population, and (3) describe associations between post-transplant cognitive impairment and quality outcome measures.

**Methods.:**

Studies in PubMed, Embase, Scopus, PsychINFO, and the Cochrane Database of Controlled Trials were included through May 2022. Inclusion criteria included (1) population – LT recipient, age ≥18 y, (2) exposure – history of cirrhosis before transplant, and (3) outcome – cognitive impairment after transplant (per validated cognitive testing). Exclusion criteria included (1) wrong study type, (2) abstract-only publication, (3) full-text unavailable, (4) wrong population, (5) wrong exposure, and (6) wrong outcome. The risk of bias was assessed using the Newcastle-Ottawa Scale and the Appraisal tool for Cross-Sectional Studies. The Grading of Recommendations, Assessment, Development, and Evaluations system was used to assess evidence certainty. Data from individual tests were categorized into six cognitive domains: attention, executive function, working memory, long-term memory, visuospatial, and language.

**Results.:**

Twenty-four studies were included covering 847 patients. Follow-up ranged from 1 mo to 1.8 y after LT. Studies had a median of 30 (interquartile range 21.5–50.5) patients. The prevalence of cognitive impairment after LT ranged from 0% to 36%. Forty-three unique cognitive tests were used, the most common being the Psychometric Hepatic Encephalopathy Score. The most frequently assessed cognitive domains were attention (10 studies) and executive function (10 studies).

**Conclusions.:**

The prevalence of cognitive impairment after LT varied across studies depending on cognitive tests utilized and follow-up duration. Attention and executive function were most impacted. Generalizability is limited due to small sample size and heterogeneous methodology. Further studies are needed to examine differences in the prevalence of post-LT cognitive impairment by etiology, risk factors, and ideal cognitive measures.

Patients with liver cirrhosis frequently experience cognitive impairment before liver transplant (LT).^1–3^ Multiple mechanisms have been proposed to contribute to pre-transplant (pre-LT) cognitive impairment, including minimal and overt hepatic encephalopathy (OHE)^1,4,5^ with or without structural and/or metabolic changes in the brain,^5,6^ chronic alcohol use,^2^ viral infection,^2^ and gut microbial dysbiosis,^7^ among others. Some of these factors, such as OHE, are thought to be reversible by LT.^8^ However, LT does not always result in a return to normal cognitive function,^2^ and if neurologic recovery takes place, the time course and degree of recovery appears to be highly variable.^2,9–11^ Postoperative complications,^12^ the stress of recovery from a major operation, and new exposure to potent immunosuppressants and antimicrobials may all contribute to cognitive impairment^13,14^ after LT. To date, there are no systematic reviews in the literature that address the subject of cognitive impairment in LT recipients with a history of cirrhosis. This paper is a systematic review that seeks to fulfill three objectives: (1) describe the prevalence of cognitive impairment in LT recipients with a history of cirrhosis; (2) describe known risk factors for cognitive impairment in LT recipients with a history of cirrhosis; and (3) describe known associations between post-LT cognitive impairment and clinical outcomes such as hospitalization, mortality, and quality of life.

## MATERIALS AND METHODS

**Figure 1.**
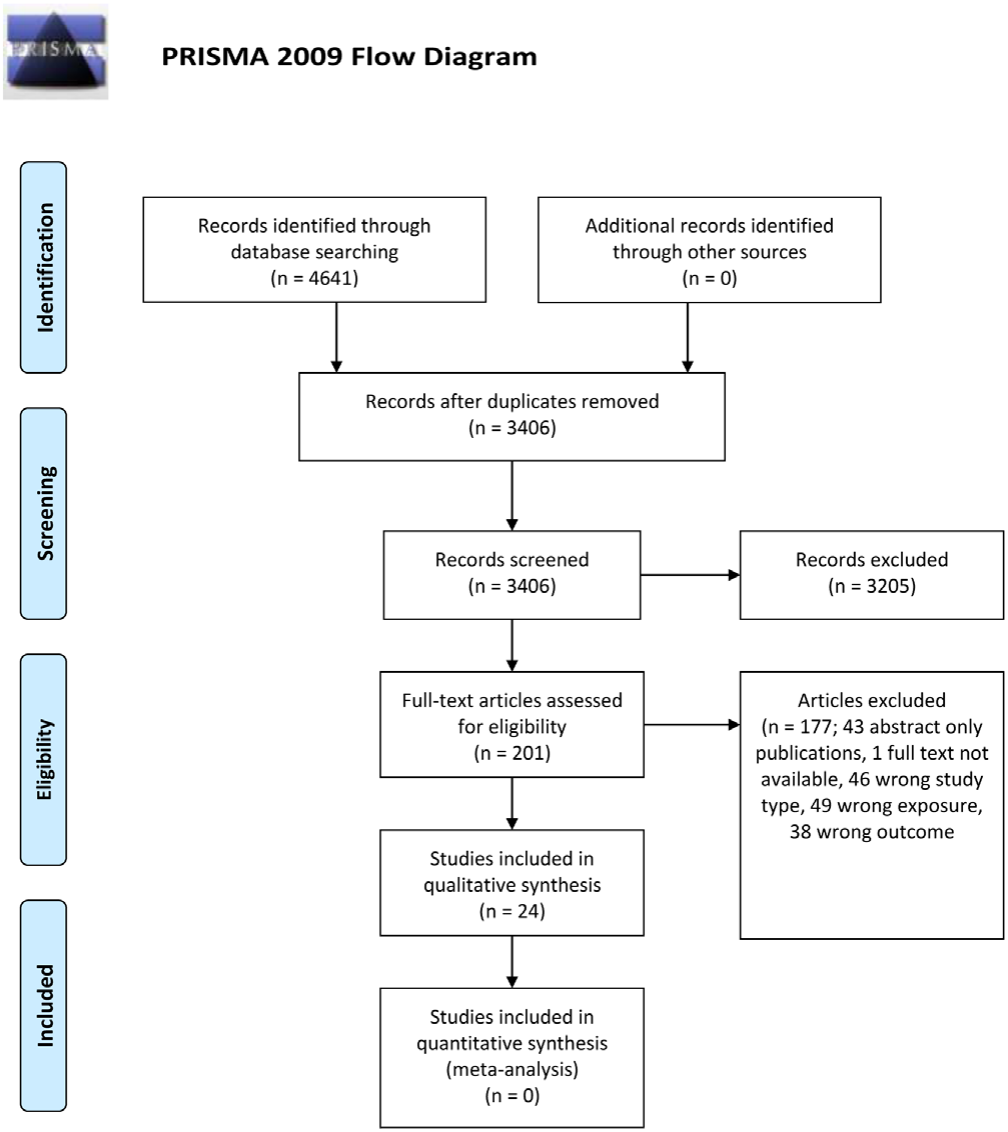
PRISMA, Preferred Reporting Items for Systematic Reviews and Meta Analyses. PRISMA format diagram describing systematic review procedure.

A systematic review of the literature was conducted according to Preferred Reporting Items for Systematic Reviews and Meta-analysis guidelines.^15^ PubMed (MEDLINE), Embase (Elsevier), Scopus (Elsevier), PsychINFO, and the Cochrane Database of Controlled Trials (Wiley) were searched from inception to May 2022. The search syntax was developed by our institution’s library scientist (M.L.B.) in conjunction with study authors and is available in our Supporting Information document. After deduplication, the search resulted in a total of 3406 unique citations that were then independently screened by three investigators (A.B.B., N.N., and T.S.). Inclusion criteria for this review included (1) population – LT recipient, age ≥18 y old, (2) exposure – history of liver cirrhosis before LT, and (3) outcome – cognitive impairment after LT (as measured by validated cognitive testing). Exclusion criteria included (1) wrong study type (case reports, case series, editorials, review papers, and textbook chapters), (2) abstract-only publication, (3) full-text unavailable, (4) wrong population (eg, age <18 y), (5) wrong exposure (indications for LT other than liver cirrhosis, eg, acute liver failure), and (6) wrong outcome (lack of validated cognitive testing, no relevant results reported). After title and abstract screening, 201 studies were selected for full-text review, which was independently performed by three investigators (A.B.B., O.M.S., and N.N.). Conflicts were resolved by mutual agreement and discussion with the principal investigator (D.P.L.) and content expert (B.B.). Twenty-four studies were ultimately included. A full visual description of our article screening workflow is available in the Preferred Reporting Items for Systematic Reviews and Meta-analysis flow diagram in Figure 1. Risk of bias assessment was manually conducted by three investigators (A.B.B., O.M.S., and K.A.L.). The Newcastle-Ottawa Scale (NOS) instrument was used to assess bias in the included cohort studies.^16^ The NOS scale for prospective cohort studies rates studies based on the parameters of selection, comparability between exposed and unexposed groups, and assessment of exposure and outcome. The maximum number of points for each category is 4 for selection, 2 for comparability, and 3 for assessment of exposure and outcome. The total score is 9 points, in which a score of 0–3 is considered as low quality, 4–6 as moderate quality, and 7–9 as high quality.^17–19^ The Appraisal Tool for Cross-Sectional Studies (AXIS) was used to assess the risk of bias in the included cross-sectional studies.^20^ AXIS is a 20-point questionnaire tool that examines the quality of studies based on study design, sample size justification, target population, measurement validity and reliability, sampling frame, and overall methods. Higher scores indicate higher study quality.^20^ The Grading of Recommendations, Assessment, Development, and Evaluations system was utilized to assess the overall certainty of evidence in the included studies.^21^ Data extraction was completed by 3 team members (A.B.B., O.M.S., and N.N.) using data tables for variables of interest which included study type, number of participants, country, age, Model for End-stage Liver Disease (MELD) score, etiology of liver cirrhosis, timing of cognitive testing, prevlance of cognitive impairment, definiton of cognitive impairment, and cognitive tests used. The data tables were used to synthesize results and write the manuscript. To facilitate synthesis, data from individual cognitive tests in different studies were mapped to six distinct cognitive domains (attention, executive function, working memory, long-term memory, visuospatial, and language). Their definitions for the purpose of this study are as follows: attention—being able to focus on relevant and important information over time; executive function—reasoning and problem-solving; working memory–conscious storage of information for adaptive use; long-term memory—longer-term storage of information; visuospatial—active skills of perception; and language—understanding, receiving, and producing language.^22,23^ However, it should be noted that some cognitive tests measure performance on multiple domains (eg, performance on the Phonemic Verbal Fluency [PVF] test is influenced by the domains of executive function and language).^24^ No automation tools or software were utilized after the initial title/abstract screening stage, during which all reviewers utilized the Rayyan Systematic Review tool.^25^ The protocol for the review was not registered. This work is supported by an NIH grant (T32DK077662), and the authors have no competing interests. All research was conducted in accordance with both the Declarations of Helsinki and Istanbul.

## RESULTS

### Study Characteristics

Of the 24 included studies, 23 (96%) were prospective cohort studies with duration of follow-up ranging from 1 mo to 1.8 y after LT.^1,2,5,7–9,26–42^ The median number of patients in the included studies was 30 (interquartile range: 21.5–50.5). Ten (42%) studies were conducted in Europe,^2,9,26,33–36,40–42^ 9 (38%) in Asia,^8,8,27–32,38,39^ and 5 (21%) in the United States.^1,3,5,7,37^ Twenty-three studies (96%) reported the mean or median age, which was between 31 and 65 y.^3,5,7–9,27–42^ Seventeen studies (71%) reported data on the etiology of liver cirrhosis^1–3,5,7–9,26,29,30,33–37,40,41^ while 8 (33%) reported the mean or median MELD score (Table 1).^1–3,5,7,9,37,40^

**TABLE 1.** Included studies

Study (type)	N [country]	Age^a^	MELD Score^a^	Etiology of cirrhosis	Timing of cognitive testing from LT	Prevalence of cognitive impairment	Definition of cognitive impairment	Cognitive tests used
Acharya et al, 2021^1^ (Prospective cohort)	61 [USA]	58 (52, 61) [pre-LT, timing not specified]	22 (16.5–24) [pre-LT, timing not specified]	34% HCV 31% EtOH 21% other 13% NASH	6 mo12 mo	22% (6 m, N = 37)16% (12 m, N = 37)	PHES ≤ −4	PHES (NCT-A, NCT-B, Digit Symbol, Serial Dotting, Line Tracing)
Ahluwalia et al, 2016^5^ (Prospective cohort)	66 [USA]	56 ± 7 [pre-LT, at listing]	21.8 ± 8.6 [pre-LT, at listing]	37% HCV 10% EtOH 13% NASH	6 ± 3 mo	21%	PHES ≤ −4	PHES (NCT-A, NCT-B, Digit Symbol, Serial Dotting, Line Tracing), block design test, ICT-T
Arria et al, 1991^37^ (Prospective cohort)	13 [USA]	39.7 ± 6.7 [pre-LT, at listing]	—	100% EtOH	12 mo	—	—	Signature Time, Finger Tapping, grooved peg board, block design test, TMT-A, TMT-B, Stroop Interference, Symbol Digit Test, Digit Span, Benton Visual Retention,
Bajaj et al, 2017^7^ (Prospective cohort)	45 [USA]	56 ± 7 [pre-LT, timing not specified]	26 ± 8 [pre-LT, timing not specified]	47% HCV 27% NASH 22% EtOH 4% other	7 ± 2 mo	36%	PHES ≤ −4	PHES (NCT-A, NCT-B, Digit Symbol, Serial Dotting, Line Tracing)
Campagna et al, 2014^2^ (Prospective cohort)	65 [Italy]	51 ± 8 [pre-LT, timing not specified]	11 ± 5 [pre-LT, timing not specified]	58% Viral 20% EtOH 14% Mixed 8% other	9–12 mo	9%	Score ≤2 SD below age and education matched controls on at least 2 of the following tests: TMT-A, TMT-B, symbol digit	TMT-A, TMT-B, Digit Span, Phonemic Verbal Fluency, symbol digit, ITM-10, ITM-30, story recall (immediate, delayed), Scan Test
Cheng et al, 2015^27^ (Prospective cohort)	12 [China]	50.9 ± 7.6 [post-LT]	—	—	1 mo	—	—	NCT-A, Digit Symbol
Cheng et al, 2017^29^ (Prospective cohort)	20 [China]	51.9 ± 6.9 [post-LT]	—	40% HCV 35% HBV 10% biliary 15% other	1 mo	—	—	NCT-A, Digit Symbol
Cheng et al, 2018^30^ (Prospective cohort)	33 total 15 (HE group) 18 (no-HE group) [China]	52.5 ± 7.8 (HE) [post-LT]52.4 ± 8.7 (no-HE) [post-LT]	—	39% HCV 36% HBV 12% biliary 12% other	1 mo	—	—	NCT-A, Digit Symbol
Cheng, Shen et al, 2021^38^ (Prospective cohort)	36 total 13 (HE group) 23 (no-HE group) [China]	49.1 ± 9.3 (HE) [post-LT]47.4 ± 9.4 (no-HE) [post-LT]	—	—	1 mo	—	—	NCT-A, Digit Symbol
Cheng, Li et al, 2021^39^ (Prospective cohort)	51 total21 (HE group)30 (no-HE group) [China]	50.5 ± 9.3 (HE) [pre-LT, timing not specified]49.6 ± 9.5 (no-HE) [pre-LT, timing not specified]	—	—	1 mo	—	—	NCT-A, Digit Symbol
Garcia-Martinez et al, 2011^40^ (Prospective cohort)	52 [Spain]	54 ± 10 [pre-LT, timing not specified]	17 ± 6 [pre-LT, timing not specified]	48% Viral46% EtOH6% other	6–12 mo	13%	Cognitive Index ≤40 (average of T-scores on all cognitive tests)	Auditory Verbal Learning, TMT-A, symbol digit, grooved peg board, COWAT, Hooper Visual Organization, judgment of line orientation
Lin, Chou et al, 2014^31^ (Prospective cohort)	28 [Taiwan]	51.1 ± 8.4 [post-LT]	—	—	6–12 mo	—	—	CASI, WCST-64, WAIS-III (Letter Number Search, picture completion, block design, Digit Symbol)
Lin, Hsu et al, 2014^32^ (Prospective Cohort)	26 [Taiwan]	51.11 ± 8.31 [post-LT]	—	—	6–12 mo	—	—	CASI, WCST-64, WAIS-III (Letter Number Search, picture completion, block design, Digit Span)
Mardini et al, 2008^26^ (Prospective Cohort)	21 [United Kingdom]	—	—	62% EtOH	16 ± 14 mo	0% (PHES)10% (CDR)	PHES ≤ −4CDR ≤ −5	PHES, CDR
Mechtcheriakov et al, 2004^33^ (Prospective Cohort)	14 [Austria]	55.6 ± 7.92 [pre-LT, timing not specified]	—	36% EtOH	21 ± 7.7 mo	—	—	Digit Symbol, TMT-A, TMT-B, RCFT-copy, SVF
O’Carroll et al, 2003^42^ (Prospective Cohort)	70 [United Kingdom]	50.4 ± 11.1 [post-LT]	—	—	12 mo	—	—	Rivermead behavioral memory test, simple reaction time, choice reaction time
Ortiz et al, 2006^34^ (Prospective cohort)	23 [Spain]	55.4 ± 11.5 [pre-LT, timing not specified]	—	87% Viral13% Other	12 mo	—	—	TMT-A, COWAT, symbol digit (oral), Auditory Verbal Learning, Grooved Pegboard, Hooper Visual Organization, judgment of line orientation
Pantiga et al, 2003^35^ (Prospective cohort)	30 [Spain]	54 ± 9 [post-LT]	—	40% EtOH10% Viral10% other	1.8 y	—	—	TMT-A, TMT-B, Digit Span, Raven’s Progressive Matrices
Rovira et al, 2007^41^ (Prospective cohort)	27 [Spain]	60 ± 9 (WML) [pre-LT, timing not specified]46 ± 11 (no-WML) [pre-LT, timing not specified]	—	44% Viral26% EtOH22% Viral + EtOH8% other	6–14 mo	3.7%	Overall Cognitive Score ≤40 (average of T-scores on all cognitive tests)	Auditory Verbal Learning, TMT-A, symbol digit, COWAT, grooved peg board, judgment of line orientation, Hooper Visual Organization
Sotil et al, 2009^3^ (Cross-sectional)	39 total25 (HE group)14 (no-HE group) [USA]	57 ± 8 (HE) [pre-LT, timing not specified]51 ± 11 (no-HE) [pre-LT, timing not specified]	24.4 ± 8.9 (HE) [pre-LT, timing not specified]16.4 ± 9.8 (no-HE) [pre-LT, timing not specified]	HE: 36% HCV,36% EtOH, 8% biliary, 28% otherNo-HE: 29% HCV, 14% EtOH, 36% biliary, 36% other	17 ± 8.1 mo (HE)18.7 ± 11 mo (no-HE)	—	—	PHES, RBANS, CFF
Tryc et al, 2014^9^ (Prospective cohort)	50 total21 (HE)29 (no-HE) [Germany]	HE: 53 (43, 58.5) [pre-LT, timing not specified]No-HE: 55 (49.5, 60) [pre-LT, timing not specified]	HE: 19 (13,25.2) [at time of LT]No-HE: 11 (8, 15.6) [at time of LT]	HE: 24% Viral, 24% EtOH, 14% biliary, 38% otherNo-HE: 17% viral, 10% EtOH, 28% biliary, 45% other	6 mo, 12 mo	6 mo: 8% (PHES), 6% (ICT-T)4% (CFF)12 mo: 0% (PHES)0% (ICT-T) 4% (CFF)	PHES ≤ −4ORAbnormal ICT-TORAbnormal CFF	PHES, RBANS, ICT-T, CFF
Vataja et al, 1994^36^ (Prospective cohort)	22 [Finland]	Mean = 38Range = 31–65 [pre-LT, timing not specified]	—	82% biliary, 18% other, 0% EtOH	6–12 mo	—	—	TMT-B, Stroop C, WAIS (Processing Speed), WMS
Zhang et al, 2017^28^ (Prospective cohort)	30 total13 (HE group)17 (no-HE group) [China]	52.3 ± 8 (HE) [post-LT]52.4 ± 9 (no-HE) [post-LT]	—	—	1 mo	—	—	NCT-A, Digit Symbol
Zhang et al, 2015^8^ (Prospective cohort)	13 [China]	50.6 ± 7.4 [post-LT]	—	0% EtOH	1 mo	—	—	NCT-A, Digit Symbol

^a^Values are formatted as median (interquartile range) or mean ± SD. — = not reported.

CASI, Cognitive Abilities Screening Instrument; CDR, cognitive drug research assessment system; CFF, Critical Flicker Frequency; COWAT, Controlled Oral Word Association Test; EtOH, alcohol; HCV, hepatitis C virus; HE, hepatic encephalopathy before LT; ICT-T, inhibitory control test; ITM-10, memory with interference task at 10 s; ITM-30, memory with interference task at 30 s; LT, liver transplant; MELD, Model for End-stage Liver Disease; MMSE, Mini-Mental State Examination; NASH, nonalcoholic steatohepatitis; NCT-A, Numbers Connection Test A; NCT-B, Numbers Connection Test B; OHE, overt hepatic encephalopathy; PHES, Psychometric Hepatic Encephalopathy Score; PVF, Phonemic Verbal Fluency; RBANS, Repeatable Battery for the Assessment of Neuropsychological Status; RCFT, Rey Complex Figure Test; SVF, Semantic Verbal Fluency; TMT-A, Trail Making Test A; TMT-B, Trail Making Test B; WAIS, Wechsler Adult Intelligence Scale; WCST-64, Wisconsin Card Sorting Test-64; WML, white matter lesion; WMS, Weschler Memory Scale.

### Study Quality

The NOS tool was used to assess the risk of bias in the 18 prospective cohort studies. One study was assessed as low quality,^36^ 16 as moderate quality,^5,7,8,27–35,38,39,41,42^ and 6 as high quality.^1,2,9,26,37,40^ The AXIS tool was used to assess the risk of bias in the 1 cross-sectional study, which met all 20 criteria for quality (Table 2). The Grading of Recommendations, Assessment, Development, and Evaluations guidelines were used to assess the certainty of evidence among included studies.^23^ The certainty of evidence was found to be low across outcomes of interest due to small sample size and heterogeneity in the included studies.

**TABLE 2.** Risk of bias assessment

Study (type)	Newcastle-Ottawa Assessment Scale for Cohort Studies (NOS)																				
	Selection								Comparability				Outcome						Total		
	1		2		3		4		5				6		7		8				
Acharya et al, 2020^1^ (Prospective cohort)	*		*		*		—		**				*		*		—		7		
Ahluwalia et al, 2016^5^ (Prospective cohort)	*		*		*		—		—				*		*		—		5		
Arria et al, 1991^37^ (Prospective cohort)	*		*		*		—		*				*		*		*		7		
Bajaj et al, 2017^7^ (Prospective cohort)	*		—		*		—		*				*		*		—		5		
Campagna et al, 2014^2^ (Prospective cohort)	*		*		*		—		**				*		*		*		8		
Cheng et al, 2015^27^ (Prospective cohort)	—		—		*		—		*				*		—		*		4		
Cheng et al, 2017^29^ (Prospective cohort)	*		—		*		—		*				*		—		*		5		
Cheng et al, 2018^30^ (Prospective cohort)	*		—		*		—		*				*		—		—		4		
Cheng, Shen et al, 2021^38^ (Prospective cohort)	*		*		*		—		*				*		—		—		5		
Cheng, Li et al, 2021^39^ (Prospective cohort)	*		*		*		—		*				*		—		—		5		
Garcia-Martinez et al, 2011^40^ (Prospective cohort)	*		*		*		—		**				*		*		—		7		
Lin, Chou et al, 2014^31^ (Prospective cohort)	—		—		*		—		*				*		*		*		5		
Lin, Hsu et al, 2014^32^ (Prospective Cohort)	*		—		*		—		*				*		*		*		6		
Mardini et al, 2008^26^ (Prospective Cohort)	*		*		*		—		*				*		*		*		7		
Mechtcheriakov et al, 2004^33^ (Prospective Cohort)	*		—		*		—		*				*		*		*		6		
O’Carroll et al, 2003^42^ (Prospective Cohort)	*		—		*		*		—				*		*		—		5		
Ortiz et al, 2006^34^ (Prospective cohort)	*		—		*		—		*				*		*		*		6		
Pantiga et al, 2003^35^ (Prospective cohort)	—		—		*		—		*				*		*		*		5		
Rovira et al, 2007^41^ (Prospective cohort)	*		—		*		—		*				*		*		*		6		
Tryc et al, 2014^9^ (Prospective cohort)	*		*		*		—		**				*		*		—		7		
Vataja et al, 1994^36^ (Prospective cohort)	—		—		*		—		—				*		*		—		3		
Zhang et al, 2017^26^ (Prospective cohort)	—		*		*		—		*				*		—		*		5		
Zhang et al, 2015^8^ (Prospective cohort)	—		—		*		—		*				*		—		*		4		
	Appraisal Tool for Cross-Sectional Studies (AXIS)																				
	Introduction	Methods										Results					Discussion		Other		Total
	1	2	3	4	5	6	7	8	9	10	11	12	13	14	15	16	17	18	19	20	
Sotil et al, 2009^3^ (Cross-sectional)	*	*	*	*	*	*	*	*	*	*	*	*	*	*	*	*	*	*	*	*	20

** = two points, * = one point, — = 0 points.

AXIS score range, 1–20 with one point possible in each category; AXIS, Appraisal tool for Cross-Sectional Studies; NOS score range, 1–9 with one point possible in each category except comparability (two points possible); NOS, Newcastle-Ottawa Scale.

### Prevalence of Cognitive Impairment

The prevalence of cognitive impairment was defined as the percentage of LT recipients meeting criteria for cognitive impairment (relative to a control group or test cutoffs based on population norms) as defined in a particular study at a particular time point. The prevalence ranged from 0% to 36% across studies with various durations of follow-up and different cognitive measures used to define cognitive impairment based on different cutoffs.^1,2,5,7,9,26,40,41^ The prevalence ranged from 4% to 36% among studies within 8 mo after transplant, whereas it ranged from 0% to 16% in studies more than 8 mo after transplant. The Psychometric Hepatic Encephalopathy Score (PHES) was the most used battery of tests among studies that reported the prevalence of cognitive impairment after LT.^1,5,7,9,26^

### Cognitive Domains

An individual cognitive test is intended to target specific domains of cognitive function. Of the 24 studies included, 19 (79%) provided data that could be mapped to 6 distinct cognitive domains (attention, executive function, working memory, long-term memory, visuospatial, and language) summarized below (Table 3).^2,3,7–9,27–39,42^

**TABLE 3.** Cognitive domains assessed

	Attention	Executive function	Working memory	Long-term memory	Visuospatial	Language
Acharya et al, 2020^1^	—	—	—	—	—	—
Ahluwalia et al, 2016^5^	—	—	—	—	—	—
Arria et al, 1991^37^	↓*	↓*	—	—	↓	—
Bajaj et al, 2017^7^	↓	↓	—	—	↓	—
Campagna et al, 2014^2^	0%–18% impaired**	11%–22% impaired**	2% impaired**	5%–8% impaired**	—	11% impaired**
Cheng et al, 2015^27^	↔	↔	—	—	—	—
Cheng et al, 2017^29^	↓	↓	—	—	—	—
Cheng et al, 2018^30^	↓ (HE, no-HE)	↓ (HE, no-HE)	—	—	—	—
Cheng, Shen et al, 2021^38^	↔ (HE, no-HE)	↔ (HE, no-HE)	—	—	—	—
Cheng, Li et al, 2021^39^	↓* (HE only)	↓* (HE only)	—	—	—	—
Garcia-Martinez et al, 2011^40^	—	—	—	—	—	—
Lin, Chou et al, 2014^31^	↔	↔*	—	↔	↔	↔
Lin, Hsu et al, 2014^32^	↓*	↔*	—	↔	↔	↔
Mardini et al, 2008^26^	—	—	—	—	—	—
Mechtcheriakov et al, 2004^33^	↔	↔	—	—	↔	↔
O’Carroll et al, 2003^42^	↓	↓	↔	↔	—	—
Ortiz et al, 2006^34^	↔	↔*	↔	↔	↔	↔
Pantiga et al, 2003^35^	↓*	↓*	—	—	—	—
Rovira et al, 2007^41^	—	—	—	—	—	—
Sotil et al, 2009^3^	↓* (HE only)	↓*(HE only)	↔ (HE, no-HE)	↔ (HE, no-HE)	↔ (HE, no-HE)	↔ (HE, no-HE)
Tryc et al, 2014^9^	↔ (HE, no-HE)	↔ (HE, no-HE)	↔ (HE, no-HE)	↔ (HE, no-HE)	↓ (no-HE only)	↔ (HE, no-HE)
Vataja et al, 1994^36^	—	↔	↔	↔	—	—
Zhang et al, 2017^28^	↓ (no-HE only)	↓ (HE, no-HE)	—	—	—	—
Zhang et al, 2015^8^	↔	↓	—	—	—	—

↔ = Normal, ↓ = impaired, — = no data, * = ≥50% of tests, ** = reported as % of participants with impaired cognitive test scores.

HE, history of hepatic encephalopathy before LT; LT, liver transplant.

#### Attention

Attention was assessed at the group level in 17 studies (71%) and the LT group showed evidence of impairment in 10 studies (59%). Campagna et al reported impaired attention in 0%–18% of LT recipients. Cognitive tests used to assess attention included the Numbers Connection Test A, Trail Making Test A, Digit Span, Cognitive Abilities Screening Instrument (CASI), Repeatable Battery for the Assessment of Neuropsychological Status (RBANS) (attention subscore), Signature Time, and Finger Tapping.

#### Executive Function

Executive function was assessed at the group level in 18 studies, and the LT group showed evidence of impairment in 10 (56%) of these studies. Campagna et al reported impaired executive function in 11%–22% of LT recipients. Cognitive tests used to assess executive function included the Numbers Connection Test B, Digit Symbol Test, Serial Dotting Test, Line Tracing Test, Trail Making Test B, Backwards Digit Span, PVF, Symbol Digit Test, Scan Test, Stroop C, CASI, Letter Number Search, Wisconsin Card Sorting Test-64, Rey Complex Figure Test, Semantic Verbal Fluency, Auditory Verbal Learning (AVL), Grooved Pegboard, Controlled Oral Word Association Test, Raven’s Progressive Matrices Test, Critical Flicker Frequency, Stroop Interference, and Wechsler Adult Intelligence Scale – Processing Speed Index.

#### Working Memory

Working memory was assessed at the group level in 6 studies, and LT recipients showed evidence of impairment in only 1 (17%) of these studies. Campagna et al reported impaired working memory in only 2% of patients after LT.^2^ Cognitive tests used to assess working memory included Immediate Story Recall Memory, AVL, RBANS (immediate memory subscore), and the Weschler Memory Scale.

#### Long-term Memory

Long-term memory was assessed at the group level in 7 studies, and LT recipients showed no evidence of impairment. Campagna et al reported impaired long-term memory in 5%–8% of LT recipients.^2^ Cognitive tests used to assess long-term memory included Memory with Interference Task at 30 s, Delayed Story Recall Memory, CASI, AVL, RBANS (delayed memory subscore), and the Weschler Memory Scale.

#### Visuospatial

The visuospatial function was assessed at the group level in 8 studies, and the LT group showed evidence of impairment in 3 studies (38%). Visuospatial tests included Serial Dotting, Line Tracing, CASI, Picture Completion, Rey Complex Figure Test, Hooper Visual Organization, Judgement of Line Orientation, RBANS (visuospatial/constructional subscore), Critical Flicker Frequency, and Benton Visual Retention.

#### Language

The language was assessed at the group level in 6 studies, and the LT group showed no evidence of impairment. Campagna et al found that 11% of patients had abnormal scores on the PVF test after LT.^2^ Tests of language used in other studies included the CASI, Semantic Verbal Fluency, Controlled Oral Word Association Test, and RBANS (language subscore).

### Risk Factors for Cognitive Impairment

Among the included studies, there was limited data available regarding risk factors for cognitive impairment after LT in patients with a history of cirrhosis. Campagna et al conducted a univariate regression analysis to evaluate age, MELD, OHE, Minimal Hepatic Encephalopathy (MHE), alcohol-associated cirrhosis, and diabetes as risk factors for cognitive impairment after LT. Since only age was found to have a trend toward significance (*P* = 0.07), a multivariate analysis was not performed.^2^

### Association With Outcomes

The included studies provided limited data on the association between post-LT cognitive impairment and clinical outcomes. In a study of 50 patients by Tryc et al, patients with a >10% decline in overall cognitive function after LT from their pre-LT baseline showed significant declines in physical, mental, and social quality of life relative to pre-LT levels.^9^ Bajaj et al showed that improvement in cognitive function after LT was associated with improved physical and mental quality of life after LT.^7^ However, neither study controlled for confounding variables (such as MELD score), and neither study directly compared the quality of life between LT recipients with and without cognitive impairment. Furthermore, no studies assessed the impact of cognitive impairment on subsequent clinical outcomes such as hospitalization and mortality.

## DISCUSSION

This is the first study to systematically synthesize existing literature on cognitive impairment in LT recipients with a history of cirrhosis. Our systematic review included 24 studies with a median of 30 patients per study, and follow-up ranging from 1 mo to 1.8 y after LT. The prevalence of cognitive impairment after LT ranged from 4% to 36% within 8 mo after LT, and from 0% to 16% more than 8 mo after LT. Attention and executive function were the most commonly examined cognitive domains, with LT recipients showing evidence of impairment in 59% and 56% of studies, respectively. According to Campagna et al, attention was impaired in 18%, and executive function was impaired in 11%–22% of LT recipients depending on the cognitive test used.^2^ Domains of working memory, long-term memory, visuospatial function, and language were examined in a few studies, most of those which reported lack of impairment post-LT.

Although included studies proposed various mechanisms for cognitive impairment after LT, most did not report data on specific risk factors. Campagna et al found a trend toward a significant association between age and cognitive impairment after LT (*P* = 0.07). Because the prevalence of cognitive impairment was low in this study (9%), it may have made it difficult to detect a statistically significant association between age and cognitive impairment.^2^ Among studies reporting prevalence of cognitive impairment, the vast majority had a study population aged 50s-60s. This could suggest that older populations with a history of cirrhosis are more predisposed to cognitive impairment post-LT. Similarly, studies with higher mean or median MELD scores (22–26)^1,5,7^ appear to have a higher prevalence of cognitive impairment after LT (21%–36%) than those with lower mean or median MELD scores (MELD range 11–19, prevalence range 8%–13%).^2,9,40^ However, studies with lower MELD scores also had population with a lower mean or median age, which is a known risk factor for cognitive impairment in the general population.^43^ Campagna et al did not find an association between post-LT cognitive impairment and OHE, MELD, MHE, alcohol-associated cirrhosis, and diabetes. Other studies identified factors associated with cognitive function after LT but did not specifically provide data on how these factors modify the risk of cognitive impairment after LT. These factors included MHE,^5^ OHE,^3,9,28–30,38–40^ alcohol-associated cirrhosis,^26,40^ immunosuppression,^9,40^ diabetes,^40^ and MELD score.^3^ A novel factor associated with post-LT cognitive function that was elucidated from the included studies was that of gut dysbiosis. Bajaj et al found that reduction in gut Proteobacteria abundance was associated with improved cognitive function after LT.^7^ Prior studies have found gut Proteobacteria abundance to be associated with endotoxemia, systemic inflammation, and negative outcomes (such as decompensated cirrhosis and increased Child-Pugh score) in chronic liver disease.^44,45^ Prior studies have also implicated the gut microbiome in the risk of post-LT infection and rejection.^46,47^ Overall, the included studies provide insufficient data to reach meaningful conclusions regarding risk factors for cognitive among LT recipients with a history of cirrhosis.

Major limitations of our review include heterogeneity in the included studies as well as small sample sizes. A major source of heterogeneity among included studies was the lack of a universally accepted definition of cognitive impairment. Out of the 8 studies that established a study-specific threshold to define cognitive impairment, 5 (63%) used a PHES score ≤−4.^1,5,7,26^ However, the PHES is only validated for the diagnosis of MHE in patients with non-alcohol-associated liver cirrhosis^48^ which limits its use in diagnosing cognitive impairment post-LT, especially in patients with a history of alcohol-associated cirrhosis. Additionally, the PHES focuses on domains frequently impaired in OHE (attention, executive function, and psychomotor speed)^49^ and would not capture impairment in other domains. The prevalence of cognitive impairment may be further underestimated by selection bias towards healthier LT recipients who are willing and able to complete a cumbersome battery of cognitive tests. For instance, some studies excluded patients with Mini-Mental State Examination scores below a certain cutoff.^5^ Therefore, larger, multi-center, longitudinal prospective studies using validated instruments to measure cognitive function across all relevant domains at regular intervals are needed. There are many crucial areas for future investigation, including establishing a standardized measure of cognitive impairment in the post-LT population, identifying LT recipients at risk for cognitive impairment, and assessing the impact of cognitive impairment on treatment adherence and outcomes.

## References

[1] Acharya C White MB Fagan A. Liver transplant is associated with sustained improvement in tandem gait and risk of falls. Dig Dis Sci. 2020;66:1360–1366. 32323075 10.1007/s10620-020-06261-y PMC7578102

[2] Campagna F Montagnese S Schiff S. Cognitive impairment and electroencephalographic alterations before and after liver transplantation: what is reversible? Liver Transpl. 2014;20:977–986. 24809329 10.1002/lt.23909

[3] Sotil EU Gottstein J Ayala E. Impact of preoperative overt hepatic encephalopathy on neurocognitive function after liver transplantation. Liver Transpl. 2009;15:184–192. 19177446 10.1002/lt.21593

[4] Acharya C Wade JB Fagan A. Overt hepatic encephalopathy impairs learning on the EncephalApp Stroop which is reversible after liver transplantation. Liver Transpl. 2017;23:1396–1403. 28885772 10.1002/lt.24864 PMC5739053

[5] Ahluwalia V Wade JB White MB. Liver transplantation significantly improves global functioning and cerebral processing. Liver Transpl. 2016;22:1379–1390. 27339647 10.1002/lt.24498 PMC5036999

[6] Patel AV Wade JB Thacker LR. Cognitive reserve is a determinant of health related quality of life in patients with cirrhosis, independent of covert hepatic encephalopathy and model for end-stage liver disease score. Clin Gastroenterol Hepatol. 2015;13:987–991. 25445772 10.1016/j.cgh.2014.09.049 PMC4404167

[7] Bajaj JS Fagan A Sikaroodi M. Liver transplant modulates gut microbial dysbiosis and cognitive function in cirrhosis. Liver Transpl. 2017;23:907–914. 28240840 10.1002/lt.24754

[8] Zhang XD Cheng Y Poon CS. Long-and short-range functional connectivity density alteration in non-alcoholic cirrhotic patients one month after liver transplantation: a resting-state fMRI study. Brain Res. 2015;1620:177–187. 25935693 10.1016/j.brainres.2015.04.046

[9] Tryc AB Pflugrad H Goldbecker A. New-onset cognitive dysfunction impairs the quality of life in patients after liver transplantation. Liver Transpl. 2014;20:807–814. 24711070 10.1002/lt.23887

[10] Garcia-Tsao G Lim JK; Members of Veterans Affairs Hepatitis C Resource Center Program. Management and treatment of patients with cirrhosis and portal hypertension: recommendations from the department of veterans affairs hepatitis c resource center program and the national hepatitis c program. Am J Gastroenterol. 2009;104:1802–1829. 19455106 10.1038/ajg.2009.191

[11] Naegele T Grodd W Viebahn R. MR imaging and (1)H spectroscopy of brain metabolites in hepatic encephalopathy: time-course of renormalization after liver transplantation. Radiology. 2000;216:683–691. 10966695 10.1148/radiology.216.3.r00se27683

[12] Guarino M Benito-Leon J Decruyenaere J.; EFNS. EFNS guidelines on management of neurological problems in liver transplantation. Eur J Neurol. 2006;13:2–9. 10.1111/j.1468-1331.2006.01353.x 16420387

[13] Vindigni SM Surawicz CM. The gut microbiome: a clinically significant player in transplantation. Expert Rev Clin Immunol. 2015;11:781–783. 26065325 10.1586/1744666X.2015.1043894

[14] Anghel D Tanasescu R Campeanu A. Neurotoxicity of immunosuppressive therapies in organ transplantation. Maedica (Bucur). 2013;8:170–175. 24371481 PMC3865126

[15] Page MJ Moher D Bossuyt PM. PRISMA 2020 explanation and elaboration: updated guidance and exemplars for reporting systematic reviews. BMJ. 2021;372:n160. 33781993 10.1136/bmj.n160 PMC8005925

[16] Wells GA Shea B O’Connell D. The Newcastle-Ottawa Scale (NOS) for assessing the quality of nonrandomised studies in meta-analyses. 2019. Available at http://www.ohri.ca/programs/clinical_epidemiology/oxford.asp. Accessed February 6, 2022.

[17] Wang Y Zhu K Dai R. Specific interleukin-1 inhibitors, specific interleukin-6 inhibitors, and GM-CSF blockades for COVID-19 (at the Edge of Sepsis): a systematic review. Front Pharmacol. 2022;12:804250. 35126138 10.3389/fphar.2021.804250 PMC8815770

[18] Zhang J Zhu H Yang W. Endoscopic stent versus diverting stoma as a bridge to surgery for obstructive colorectal cancer: a systematic review and meta-analysis [published online ahead of print, 2022 Jun 6]. Langenbecks Arch Surg. 2022;407:3275–3285. 35666309 10.1007/s00423-022-02517-5 PMC9722815

[19] Huang X. Cotton dust exposure and risk of lung cancer: a meta-analysis of observational studies. Medicine (Baltim). 2020;99:e19565. 10.1097/MD.0000000000019565 PMC7220483 32243374

[20] Downes MJ Brennan ML Williams HC. Development of a critical appraisal tool to assess the quality of cross-sectional studies (AXIS). BMJ Open. 2016;6:e011458e011458. 10.1136/bmjopen-2016-011458 PMC5168618 27932337

[21] Guyatt GH Oxman AD Vist GE.; GRADE Working Group. GRADE: an emerging consensus on rating quality of evidence and strength of recommendations. BMJ. 2008;336:924–926. 18436948 10.1136/bmj.39489.470347.AD PMC2335261

[22] Harvey PD. Domains of cognition and their assessment. Dialogues Clin Neurosci. 2019;21:227–237. 31749647 10.31887/DCNS.2019.21.3/pharvey PMC6829170

[23] Guyatt GH Oxman AD Schünemann HJ. GRADE guidelines: a new series of articles in the journal of clinical epidemiology. J Clin Epidemiol. 2011;64:380–382. 21185693 10.1016/j.jclinepi.2010.09.011

[24] Shao Z Janse E Visser K. What do verbal fluency tasks measure? Predictors of verbal fluency performance in older adults. Front Psychol. 2014;5:772. 25101034 10.3389/fpsyg.2014.00772 PMC4106453

[25] Ouzzani M Hammady H Fedorowicz Z. Rayyan-a web and mobile app for systematic reviews. Syst Rev. 2016;5:210. 27919275 10.1186/s13643-016-0384-4 PMC5139140

[26] Mardini H Saxby BK Record CO. Computerized psychometric testing in minimal encephalopathy and modulation by nitrogen challenge and liver transplant. Gastroenterology. 2008;135:1582–1590. 18647604 10.1053/j.gastro.2008.06.043

[27] Cheng Y Huang L Zhang X. Liver transplantation nearly normalizes brain spontaneous activity and cognitive function at 1 month: a resting-state functional MRI study. Metab Brain Dis. 2015;30:979–988. 25703240 10.1007/s11011-015-9657-1

[28] Zhang G Cheng Y Shen W. The short-term effect of liver transplantation on the low-frequency fluctuation of brain activity in cirrhotic patients with and without overt hepatic encephalopathy. Brain Imaging Behav. 2017;11:1849–1861. 27917450 10.1007/s11682-016-9659-6

[29] Cheng Y Huang LX Zhang L. Longitudinal intrinsic brain activity changes in cirrhotic patients before and one month after liver transplantation. Korean J Radiol. 2017;18:370–377. 28246517 10.3348/kjr.2017.18.2.370 PMC5313525

[30] Cheng Y Zhang G Shen W. Impact of previous episodes of hepatic encephalopathy on short-term brain function recovery after liver transplantation: a functional connectivity strength study. Metab Brain Dis. 2018;33:237–249. 29170933 10.1007/s11011-017-0155-5

[31] Lin WC Chou KH Chen CL. Longitudinal brain white matter alterations in minimal hepatic encephalopathy before and after liver transplantation. PLoS One. 2014;9:e105887. 25166619 10.1371/journal.pone.0105887 PMC4148329

[32] Lin WC Hsu TW Chen CL. Reestablishing brain networks in patients without overt hepatic encephalopathy after liver transplantation. J Cereb Blood Flow Metab. 2014;34:1877–1886. 25227607 10.1038/jcbfm.2014.143 PMC4269738

[33] Mechtcheriakov S Graziadei IW Mattedi M. Incomplete improvement of visuo-motor deficits in patients with minimal hepatic encephalopathy after liver transplantation. Liver Transpl. 2004;10:77–83. 14755782 10.1002/lt.20009

[34] Ortiz M Cordoba J Jacas C. Neuropsychological abnormalities in cirrhosis include learning impairment. J Hepatol. 2006;44:104–110. 16169117 10.1016/j.jhep.2005.06.013

[35] Pantiga C Rodrigo LR Cuesta M. Cognitive deficits in patients with hepatic cirrhosis and in liver transplant recipients. J Neuropsychiatry Clin Neurosci. 2003;15:84–89. 12556577 10.1176/jnp.15.1.84

[36] Vataja R Muuronen A Hillbom M. Neurological recovery after liver transplantation: a prospective study of 22 patients. Transpl Int. 1994;7(Suppl 1):S50–S51. 11271292 10.1111/j.1432-2277.1994.tb01309.x

[37] Arria AM Tarter RE Starzl TE. Improvement in cognitive functioning of alcoholics following orthotopic liver transplantation. Alcohol Clin Exp Res. 1991;15:956–962. 1789392 10.1111/j.1530-0277.1991.tb05195.x PMC3022503

[38] Cheng Y Shen W Xu J. Neuromarkers from whole-brain functional connectivity reveal the cognitive recovery scheme for overt hepatic encephalopathy after liver transplantation. eNeuro. 2021;8:ENEURO.0114–ENEU21.2021. 34376523 10.1523/ENEURO.0114-21.2021 PMC8376297

[39] Cheng Y Li JL Zhou JM. Renormalization of thalamic sub-regional functional connectivity contributes to improvement of cognitive function after liver transplantation in cirrhotic patients with overt hepatic encephalopathy. Korean J Radiol. 2021;22:2052–2061. 34564958 10.3348/kjr.2020.1432 PMC8628146

[40] Garcia-Martinez R Rovira A Alonso J. Hepatic encephalopathy is associated with posttransplant cognitive function and brain volume. Liver Transpl. 2011;17:38–46. 21254343 10.1002/lt.22197

[41] Rovira A Mínguez B Aymerich FX. Decreased white matter lesion volume and improved cognitive function after liver transplantation. Hepatology. 2007;46:1485–1490. 17929307 10.1002/hep.21911

[42] O’Carroll RE Couston M Cossar J. Psychological outcome and quality of life following liver transplantation: a prospective, national, single-center study. Liver Transpl. 2003;9:712–720. 12827558 10.1053/jlts.2003.50138

[43] (CDC) CfDCaP. Cognitive impairment: a call for action, now. 2011. Available at https://www.cdc.gov/aging/pdf/cognitive_impairment/cogimp_poilicy_final.pdf. Accessed July 2, 2021.

[44] Bajaj JS Betrapally NS Gillevet PM. Decompensated cirrhosis and microbiome interpretation. Nature. 2015;525:E1–E2. 26381988 10.1038/nature14851 PMC4617624

[45] Chen Y Yang F Lu H. Characterization of fecal microbial communities in patients with liver cirrhosis. Hepatology. 2011;54:562–572. 21574172 10.1002/hep.24423

[46] Xie Y Luo Z Li Z. Structural shifts of fecal microbial communities in rats with acute rejection after liver transplantation. Microb Ecol. 2012;64:546–554. 22430504 10.1007/s00248-012-0030-1

[47] Xie YR Liu SL Liu X. Intestinal microbiota and innate immunity-related gene alteration in cirrhotic rats with liver transplantation. Transplant Proc. 2011;43:3973–3979. 22172882 10.1016/j.transproceed.2011.08.113

[48] Weissenborn K Ennen JC Schomerus H. Neuropsychological characterization of hepatic encephalopathy. J Hepatol. 2001;34:768–773. 11434627 10.1016/s0168-8278(01)00026-5

[49] Duarte-Rojo A Estradas J Hernández-Ramos R. Validation of the Psychometric Hepatic Encephalopathy Score (PHES) for identifying patients with minimal hepatic encephalopathy. Dig Dis Sci. 2011;56:3014–3023. 21461913 10.1007/s10620-011-1684-0

